# The workforce trends of physician assistants in Iowa (1995-2015)

**DOI:** 10.1371/journal.pone.0204813

**Published:** 2018-10-08

**Authors:** Thomas S. Gruca, Gregory C. Nelson, Linda Thiesen, David P. Asprey, Sean G. Young

**Affiliations:** 1 Tippie College of Business, University of Iowa, Iowa City, IA, United States of America; 2 Office of Statewide Clinical Education Programs, Carver College of Medicine, University of Iowa, Iowa City, IA, United States of America; 3 Department of Physician Assistant Studies and Services, Carver College of Medicine, University of Iowa, Iowa City, IA, United States of America; 4 Department of Environmental and Occupational Health, University of Arkansas for Medical Sciences, Little Rock, AR, United States of America; Duke University, UNITED STATES

## Abstract

**Background:**

Physician assistants are expected to have an important role in providing both primary and specialty care. Iowa has a large rural (and aging) population and faces challenges to provide equitable access to care. This study examined changes in the Iowa physician assistant workforce (1995–2015) focusing on practice setting (primary v. subspecialty care) and geographic location (rural/urban, Health Professional Shortage Area). Documenting their current locations and service in HPSAs for primary care will help health planners track future changes.

**Methods:**

Data from 1995–2015 from the Iowa Health Professions Inventory (Office of Statewide Clinical Education Programs, Carver College of Medicine, University of Iowa) were combined with US census data on rural location and HPSA status. SPSS was used to compare Iowa and national data. Growth trends were analyzed using joinpoint regression.

**Results:**

The overall Iowa physician assistant workforce increased 161% between 1995 and 2015. In 2015, more than two-thirds (71%) were female and more than 30% practiced in rural counties. The average annual growth rate of primary care PAs (per 100,000 population) was significantly higher in the periods from 1995–1997 and 1997–2001 (22.4% and 7.4% respectively) than in period from 2001–2015 (3.8%). By 2015, 56% of Iowa’s physician assistants practiced in primary care (versus 29.6% nationally). Of these, 44% of primary care physician assistants in Iowa practiced in counties, geographic locations or worksites designated as Health Professional Shortage Areas for primary care.

**Conclusions:**

A high proportion of Iowa’s physician assistant workforce practiced in primary care and many served patients in Health Professional Shortage Areas. The number of physician assistants in Iowa will continue to grow and serve an important role in providing access to health care, particularly to rural Iowans.

## Introduction

As a state with a large and aging rural population, Iowa faces many challenges in providing equitable access to primary care to all of its citizens. The problems associated with the coming shortage of primary care physicians are amplified in a rural state [1]. In Iowa today, the situation is already of concern. Sixty-one of the 99 counties in Iowa are designated in whole (45) or part (16) to be Health Professional Shortage Areas (HPSA) for primary care [2].

One proposed approach to expanding access to primary care involves the establishment of multi-provider teams integrating primary care physicians, physician assistants (PAs) and advanced registered nurse practitioners (ARNPs) [3–4]. Such teams of primary care providers could provide a workforce capable of meeting the needs of the state. This strategy has motivated recent state-level studies of PA workforce trends in Indiana [5] and North Carolina [6].

In discussing the roles of PAs and ARNPs in meeting the needs of rural primary care patients, policy analysts seem to imply that these non-physician providers are interchangeable. However, as in almost all other states, Iowa requires physician supervision of PAs [7]. Iowa physicians are limited to supervising no more than five PAs at any given point in time. In contrast, ARNPs in Iowa have no requirement for a relationship with a physician in order to practice [7]. The question of whether PAs and ARNPs are substitutes for one another is a controversial one [6, 8].

While PAs are viewed as a part of the solution to the shortage of primary care physicians, they are also seen as key to meeting the growing demand for subspecialty care. In many medical subspecialties, there is an expected shortage of providers in the near future [9–12]. Unfortunately, this comes at a time when the demand for subspecialist medical care is expected to surge with the aging of the U.S. population [13]. Workforce studies in cardiology [9], medical oncology [10], and otolaryngology [11] advocate expanding the roles of advanced practice providers, i.e. PAs and ARNPs, to help deal with coming shortages of subspecialist physicians in those fields. For example, in urology, patients are already facing a shortage of providers [12]. Consequently, urologists are being strongly encouraged to integrate more PAs and ARNPs into their practices [12]. In Iowa, the number of urology procedures performed by PAs and ARNPs grew 56% between 2010 and 2013 [14]. The coming increase in demand for PAs in subspecialty care will be an important factor shaping the PA workforce in Iowa and throughout the U.S.

In addition to the evolving demand for PAs in general, the supply conditions in Iowa are undergoing a major change. Until 2014, there were only two PA training programs in Iowa. The older program at the University of Iowa graduated its first class in 1974. A second program at Des Moines University graduated its first class in 1981. A new program in Davenport (St. Ambrose University) graduated its first class in December 2016. A second new program located in Dubuque (University of Dubuque) graduated its first class in 2018.

Currently, the University of Iowa and Des Moines University PA programs graduate 25 and 50 students each year respectively. The addition of 30 graduates from the St. Ambrose University program and the 25 graduates from the University of Dubuque represents a 73% increase, from 75 to 130, in the annual supply of new PAs trained in Iowa once all existing programs are producing graduates.

With the many changes facing the Iowa PA workforce, there is a need for a comprehensive baseline study. The analysis presented here will examine growth and attrition patterns, demographics, primary/subspecialty focus, urban/rural distribution and location within a HPSA for primary care. This study provides detailed information to state planners, health care systems, PA programs, graduates and other stakeholders about the past and current state of the PA workforce in Iowa.

## Study data and methods

### Data

The major data source for this study was the Iowa Health Professions Inventory, maintained by the Office of Statewide Clinical Education Programs, a division of the Carver College of Medicine, University of Iowa. Statewide tracking of the physician workforce in Iowa began in 1974. Currently, the Iowa Health Professions Inventory consists of complete inventories of dentists, pharmacists, PAs (starting in 1995) and Advanced Practice Nurses (hereafter APNs) (starting in 1998). The database includes demographic, educational and professional information for these and other licensed health care professionals in Iowa. Data are collected from a number of sources including a twice-yearly telephone census of all locations in Iowa that employ a health care professional.

The data available for physician assistants in Iowa includes birth year, sex, specialty, worksite location (address level), education (school, school location, graduation year), and date of first starting work in Iowa. The practice specialty of the PA was reported by the respondent for the PA’s worksite. An external advisory committee meets yearly with the staff of the Office of Statewide Clinical Education Programs to review the state of the PA workforce and changes in the past year. The advisory committee also provides input to improve the PA tracking system over time.

Following the designation used to identify Health Profession Shortage Areas (HPSAs) for primary care, we defined primary care practice as family medicine, general internal medicine, general pediatrics and obstetrics/gynecology. To track the overall growth of primary care PAs in Iowa over time, we used this broader definition of primary care. However, when we focused on the geographic locations of PAs, APNs and primary care physicians, we used a more restrictive definition of primary care provider. For example, we did not include those providers who saw patients in a federal health care institution (e.g., VA hospital or clinic). Similarly, we did not include those providers whose worksite was considered an urgent care clinic.

To determine the practice specialty of APNs in 2015, we first extracted all records for those APNs designated as Family Nurse Practitioners (NPs), Pediatric NPs, Adult NPs, Obstetrics-Gynecology/Women’s Health NPs and Certified Nurse Midwives. Using data on practice setting, we eliminated all nurses in hospitalist, administrative or teaching roles as well as those working for federal hospitals or specialty clinics within hospitals. Finally, using data from each worksite, we screened out nurses working in urgent care centers or for specialty medical practices, e.g. cardiology.

We similarly excluded physicians with a self-reported specialty in primary care if they were employed in administration, as a hospitalist or in an urgent care center. Physicians working for federal hospitals or in a non-clinical role for a state agency were also excluded.

The data presented here is based on cross-sectional queries as of 12/31 of the target year.

Each county in Iowa was classified using the 2013 Rural Urban Continuum Codes [15]. Urban counties were subdivided into three categories based on population size: > 1 million, 250,000–1 million and less than 250,000. Non-metropolitan counties were subdivided by their adjacency to a metropolitan (urban) county. Those adjacent to a metropolitan county were further subdivided into those with an urban population greater than 20,000, between 2,500 and 20,000 and less than 2,500. Counties that are not adjacent to a metropolitan county were similarly classified based on sizes of their urban population, i.e., greater than 20,000, between 2,500 and 20,000 and less than 2,500.

The RUCC system distinguishes between rural counties that may be of comparable size but may have very different access to healthcare professionals in nearby urban counties depending on whether or not they are directly adjacent to an urban county. Prior research on the location of PAs in Iowa [16] ignored this importance distinction between different types of rural counties (non/adjacent to an urban county).

County and state-level population data were obtained from the US Census. Data from the Health Resources and Service Administration was used to identify counties and practice locations classified as primary care HPSAs [17].

### Analysis

Statistical tests of differences in means and proportions were conducted using SPSS Version 23.0. Primary care providers’ practice locations were geocoded using ArcMap 10.6.

Growth trends in the total number of PAs (per 100,000 in population) and number of PAs in primary care were modeled using joinpoint regression [18]. This method attempts to fit a set of straight lines (with different slopes) to trends in a time series of data. We restricted the program to those solutions where there were at least 3 annual observations between joinpoints or between a joinpoint and either end of the data series. Since there are 22 years of data in this study, we restricted the software to consider only 3 joinpoints. Before the models were estimated, the dependent variables were log transformed.

For model selection, we used the Bayesian Information Criterion 3 method. This metric employs a harsher penalty for addition parameters than the usual BIC model. The BIC3 for a joinpoint regression with k joinpoints and N observations is given by: BIC3(k) = ln(SSE(k)/N) + ((3K+2)/N)*ln(N) where SSE(k) is the sum of squared errors for a k-joinpoint regression model. The results from the model with the minimum BIC3 are reported below.

## Results

### Workforce growth and attrition, 1995–2015

**Fig 1:**
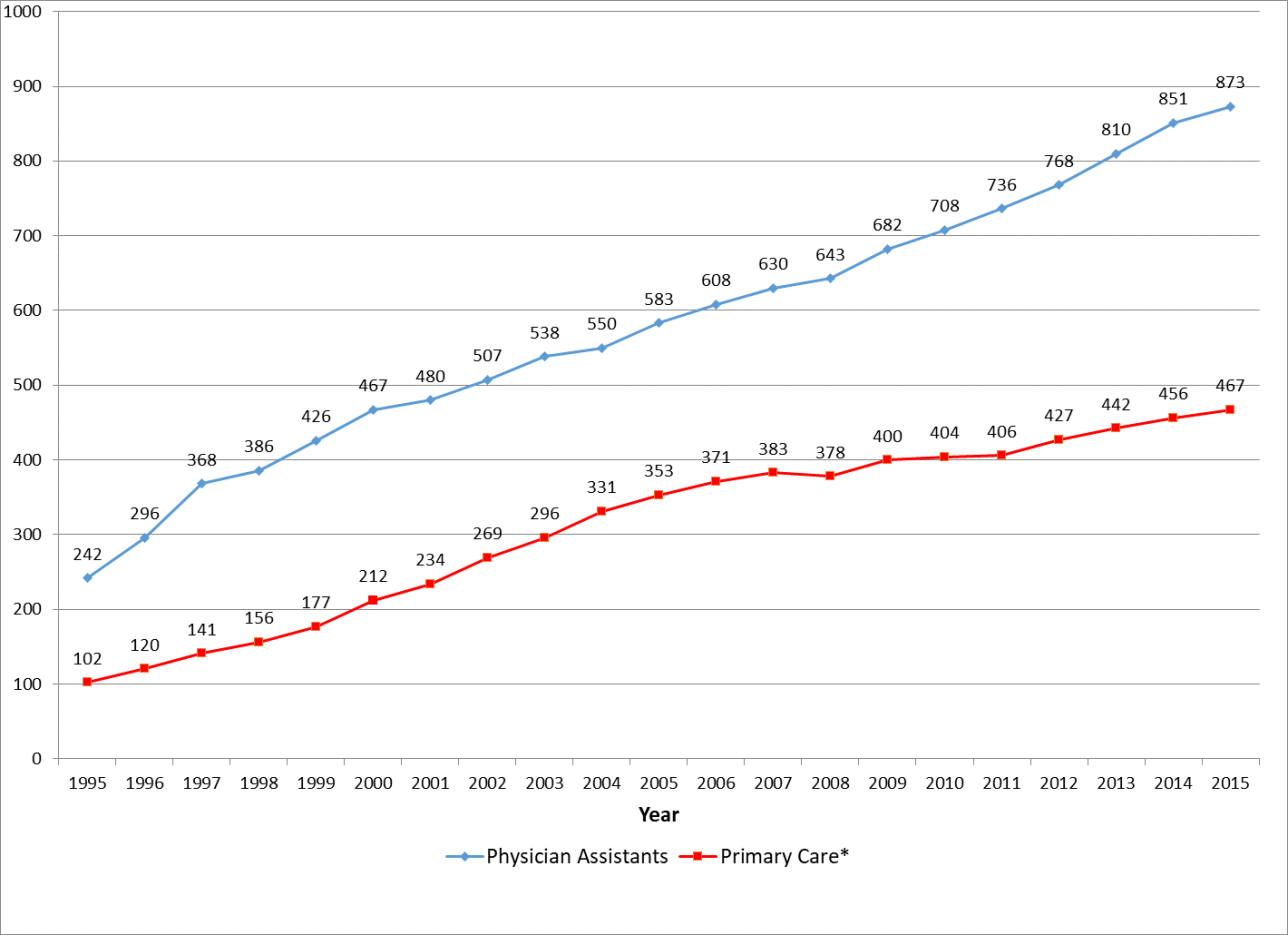
Iowa physician assistant supply: 1995–2015.

The number of PAs practicing in Iowa has grown rapidly since 1995. The total number of PAs increased 161% from 242 at the end of 1995 to 873 by the end of 2015 (Fig 1). By 2015, there were 28 PAs per 100,000 residents in Iowa. This is lower than the overall US average of 33.9 [19].

We examined the comparable growth rates for PAs, physicians and advance practice nurses from 1998 until 2017. We chose this period since data was available for all three practitioner groups. The overall growth in the PA workforce during these 17 years was 126% which was higher than the overall growth rate for physicians (60%) but lower than the overall growth rate for APNs (275%).

The joinpoint analysis of the number of PAs per 100,000 in population found that growth rates have been slowing over time (See Table 1). The average percentage change (APC) from 1995–1997 was 22.4% which was significantly higher than the APC of 7.4% from 1997–2001 (t = -5.42, p < 0.000). The APC of 3.8% in the years 2001–2015 was significantly lower than that of the period 1997–2001 (t = -4.16, p <0.002).

**Table 1:** Joinpoint^a^ analysis results for significant change in trend in total PAs per 100,000 population and primary care PAs per 100,000 population in Iowa: 1995–2015.

Total PAs per 100,000 population	Years	APC^b^ (95% CI^c^)
	1995–1997	22.4 (16.5, 28.5)
	1997–2001	7.4 (5.5, 9.3)
	2001–2015	3.8 (3.6, 3.9)
Primary care PAs per 100,000 in population	1995–2002	14.4 (13.3, 15.3)
	2002–2005	10 (4.2, 16.5)
	2005–2015	2.2 (1.9, 2.5)

a Joinpoint Regression Program, version 4.6.0.0—April 2018; Statistical Methodology and Applications Branch, Surveillance Research Program, National Cancer Institute. Bethesda MD

b APC = annual percentage change in PAs per 100,000 in population

c CI = confidence interval.

As noted above, a PA is considered to be in primary care if he or she is reported to provide care in family medicine, general internal medicine, general pediatrics or obstetrics/gynecology. Using this definition, the number of PAs in primary care grew from 103 in 1995 to 485 in 2015, an increase of 371%. The growth rate in the primary care PA workforce has varied over time. A joinpoint analysis (Table 1) shows that the APC for PAs in primary care was 14.4% from 1995–2002. In the period 2002–2005, the APC fell to 10%. This is significantly higher than the APC of 2.2% in the 2005–2015 period (t = -2.91, p < 0.02).

While the growth in Iowa’s PA workforce presented in Fig 1 appears to be relatively continuous, the reality was somewhat different. While a number of PAs entered the workforce each year, there also was attrition occurring for various reasons. (The yearly attrition figures for the years 1995–2015 are presented in S1 Table).

Since 1995, an average of 32.6 PAs left the Iowa workforce (average annual attrition rate of 5%). With the growth in the size of the PA workforce, the average turnover has been higher in the most recent five years (42.3 –average annual attrition rate of 6%). The most common reason was relocation (40%) followed by a miscellaneous category (36%) that included numerous factors such as loss of license, military service as well as those whose reason for leaving is unknown. Professional inactivity (10%) and retirement (9%) were the next most common sources of turnover.

Between 1995 and 2015, the average retirement age for PAs in Iowa was 64 years old.

### Workforce demographics

In 1995, 58% of PAs in Iowa were women and 42% were men. By 2015, the percentage of women increased to 71%. The comparable proportion of women in the national [19] PA workforce in 2015 was 67.2%. The proportion of women was higher in Iowa than in the national PA workforce (Z = 2.39, p < 0.02, 95% CI 67.87, 73.99).

**Fig 2:**
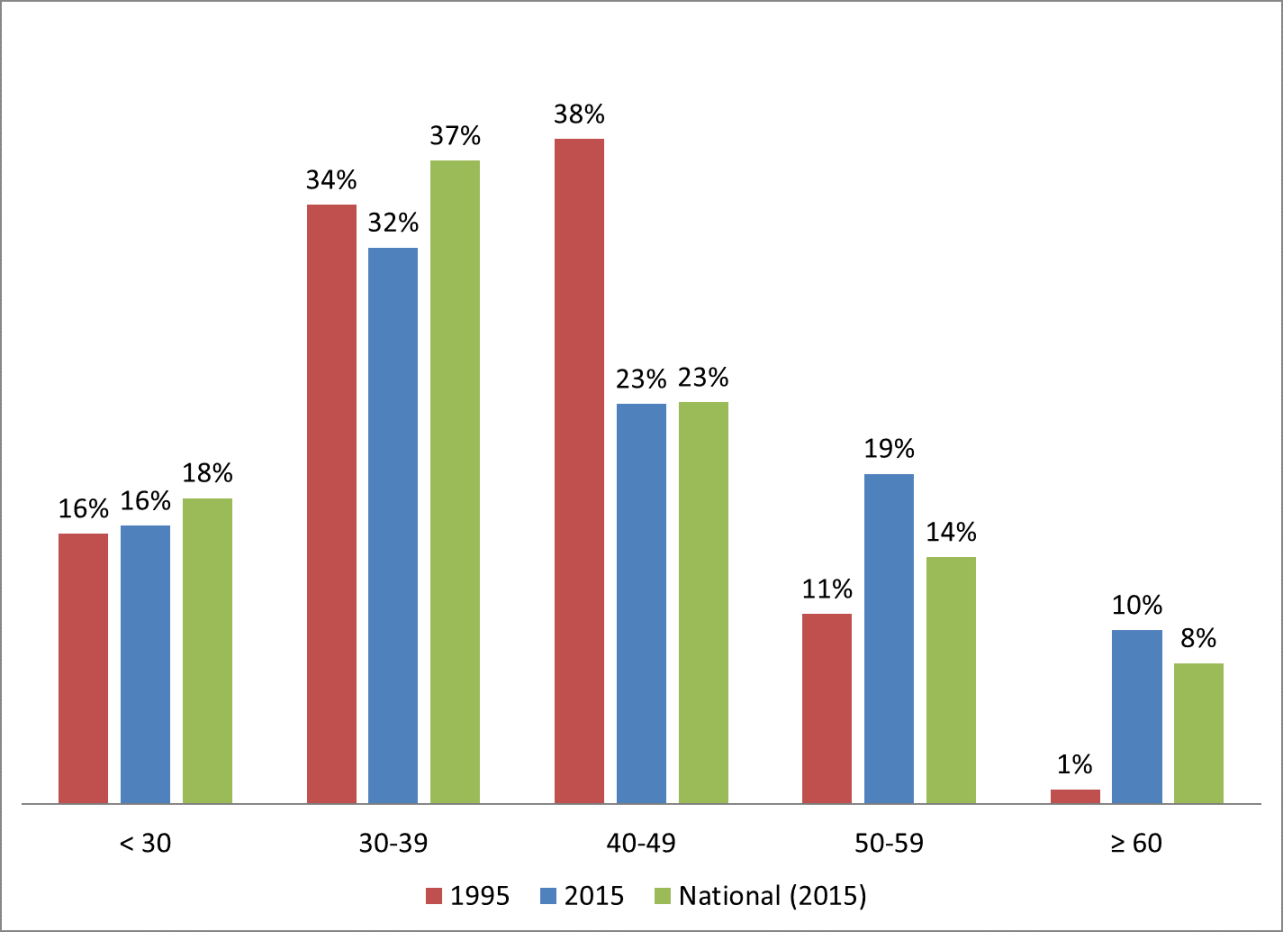
Age distribution of Iowa physician assistants in 1995 and 2015.

In 1995, the average age of PAs in Iowa was 39.9 years (median = 40). By 2015, the average age increased to 42 years of age. The median in 2015 remained at 40 years of age. The distribution of PAs by age in 1995 and 2015 is presented in Fig 2. While the average age in Iowa had not changed substantially, the distribution reflects an increasingly aged PA workforce. For example, in 1995, 11% of PAs in Iowa were over 50. By 2015, that figure rose to 29% of the much larger workforce. This was significantly higher than the comparable figure of 22.3% for the national [19] PA workforce (Z = 4.76, p < 0.001, 95% CI 26.0, 32.1).

### Workforce training and retention

More than half (54%) of Iowa’s existing PA workforce trained in Iowa. Of those, 30% trained at Des Moines University while the remaining 24% graduated from the University of Iowa’s program. An additional 22% of the workforce trained in adjoining states (IL, KS, MN, MO, NE, SD, and WI).

Of the 728 PAs trained by the University of Iowa between 1983 and 2015, 187 were practicing in Iowa in 2015. This reflects an overall retention rate of 26%. During that same period, 1024 PAs trained at Des Moines University. Of those, a total of 252 PAs were practicing in Iowa in 2015 reflecting a retention rate of 25%.

### Practice focus

**Fig 3:**
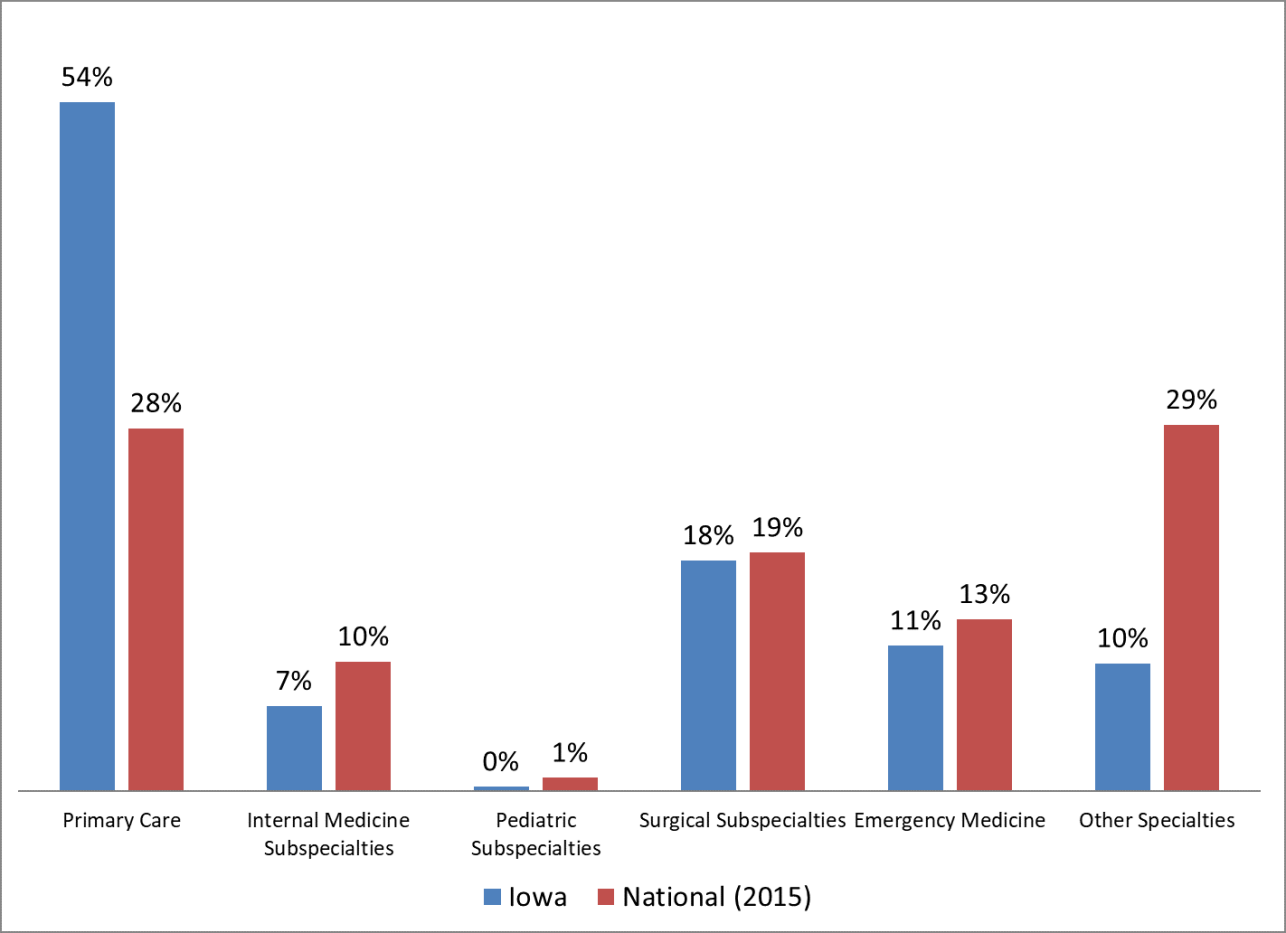
Practice specialty of Iowa physician assistants in 2015.

Using the definitions from the National Commission on Certification of Physician Assistants [19], we classified all PAs in Iowa into one of 6 major areas: primary care (including obstetrics/gynecology), internal medicine subspecialties, pediatric subspecialties, surgical subspecialties, emergency medicine and other. The results for 2015 are presented in Fig 3.

In 2015, 56% of all PAs in Iowa are in primary care which included family medicine, general internal medicine, general pediatrics, and obstetrics/gynecology. This is substantially higher than the national [19] average of 30% (Z = 16.8, p < 0.0001, 95% CI 52.6, 59.3). As would be expected, the percentage of Iowa PAs in other classifications is lower than the national norms.

### Geographic distribution

**Fig 4:**
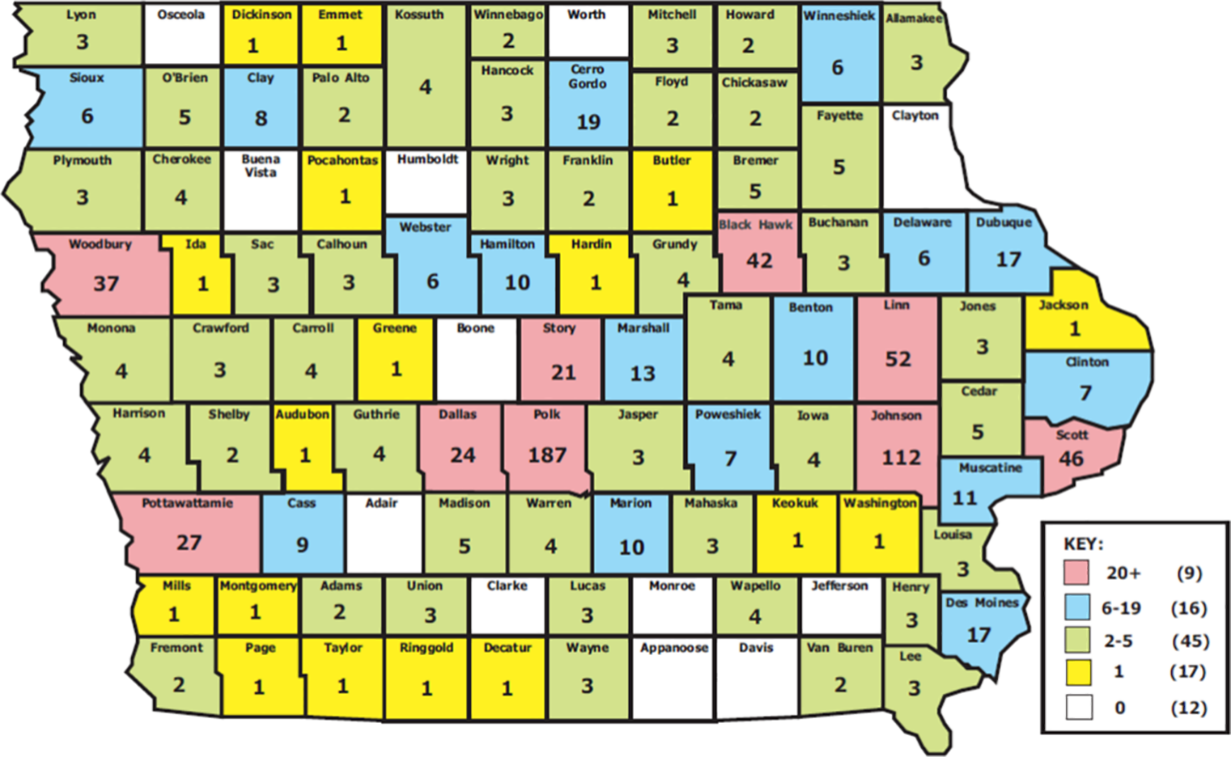
Total Iowa physician assistants by county in 2015.

PAs were employed in 88 of Iowa’s 99 counties (Fig 4). Four counties–Polk (n = 180), Johnson (n = 112), Linn (n = 52) and Scott (n = 46)–account for 45% of all PAs in Iowa.

Using the 2013 Rural Urban Continuum Codes [13], we classified all of the counties where PAs were employed in Iowa in 2015. We have also included the distribution of primary care PAs and the proportion of Iowa’s population living in each type of county. The results are presented in Table 2.

**Table 2:** Distribution of physician assistants in Iowa by rural/urban continuum code^a^ (2015).

	Metro (%)			Non-metro, adjacent to metro (%)			Non-metro, not adjacent to metro area (%)		
Population	> 1M	250K-1M	< 250	> 20K	2.5K-20K	<2.5K	>20K	2.5K-20K	< 2.5K
**National PAs**^**b**^	48.5	24.5	11.9	4.4	3.6	0.7	2.5	3.0	1.0
**Iowa PAs (n = 873)**	0	41.8	27.7	3.6	10.3	1.6	5.6	7.4	1.9
**Iowa Primary Care PAs (n = 423)**	0	35.0	21.5	3.8	17.5	3.3	3.8	11.1	4.0
**Iowa population percentage (2015)**^**c**^	0	37.4	20.9	4.3	13.8	3.0	6.2	11.7	2.6
**Iowa Primary Care PA** **Index**	0	94	103	88	127	110	61	95	154
**Iowa Primary Care APNs (n = 857)**	0	28.7	20.1	3.6	16.3	4.3	9.1	13.8	4.1
**Iowa Primary Care APN Index**	0	77	96	84	118	143	147	118	158
**Iowa Primary Care Physicians (n = 1979)**	0	36.8	27.5	3.3	11.0	1.4	7.3	11.1	1.6
**Iowa Primary Care Physician Index**	0	98	132	77	80	47	118	95	62

a USDA Economic Research Service. 2013 Rural-Urban Continuum Codes

b Source: National physician assistant census report 2009, Research and Technology Services, American Academy of Physician Assistants [20].

^C^ US Census Bureau’s American Community Survey [21]

As of 2015, some 70% of Iowa PAs practiced in urban areas, which is substantially lower than the comparable national [20] proportion of 85% (Z = 12.86, p < 0.0001, 95% CI 66.3, 72.5). This implies that a significantly higher proportion of PAs in Iowa were employed in non-metro counties (30%) than at the national level (15%). Furthermore, in Iowa, PAs were more likely (14.9% in Iowa vs. 6.5 nationally [20]) to practice in more rural counties, i.e., non-metro, nonadjacent to metro areas (Z = 10.07, p < 0.0001, 95% CI 12.6, 17.4). It should be noted that the geographic distribution of the overall PA workforce in Iowa is consistent with the distribution of Iowa’s population. For example, only 58% of Iowans lived in metropolitan counties in 2015.

To better assess the impact of PAs on providing care to all Iowans, those PAs in primary care (family medicine, general internal medicine, general pediatrics and obstetrics/gynecology) were analyzed separately from those in other practice areas. As noted above, we excluded those primary care PAs who were working in VA facilities or urgent care clinics. This reduced the sample size to n = 423 PAs.

For each type of county, we computed the ratio of the proportion of Iowa’s primary care PAs in that type of county to the proportion of Iowa’s population in that category of county. We multiplied the resulting figure by 100 to produce an index where 100 indicates a ratio consistent with the population share. A comparable index for primary care physicians and primary care APNs were computed using the same population data. These results are included in Table 2.

Primary care PAs were practicing in proportion to the population in larger metro counties (Index = 94) while primary care physicians were over-represented (Index = 132) in metro counties with smaller (< 250K) cities. This latter result was likely due to the large number of family medicine physicians employed in Johnson County, which is home to the state’s only academic medical center.

Primary care PAs were practicing in numbers roughly proportion to the population in non-metro counties adjacent to metro areas with a large (> 20,000) city (Index = 88). These counties similarly served by primary care APNs (Index = 84). Primary care physicians (Index = 77) were underrepresented.

In contrast, primary care PAs were over-represented in non-metro counties adjacent to metro areas with mid-sized (2,500–20,000) cities (Index = 127). Primary care APNs were over-represented in these counties (Index = 118) while primary care physicians are under-represented (Index = 80).

In non-metro counties (adjacent to metro counties) with small metro populations (<2.5k), primary care PAs were proportionately represented (Index = 110). Primary care APNs were highly over-represented (Index = 143) while primary care physicians were very under-represented (Index = 47).

Primary care PAs are highly over-represented (Index = 154) in the non-metro, non-adjacent counties with the smallest metro areas (< 2.5K). This also true of primary care APNs (Index = 158). Both of these non-physician practitioners seem to be substitutes for primary care physicians Primary care physicians were significantly under-represented (Index = 62).

It is interesting to note that primary care PAs are underrepresented (Index = 61) in the non-metro, nonadjacent counties with the largest metro populations (>20K). These 5 counties have a high proportion of PAs overall (5.6% versus 6.2% population share). The presence of a high number of PAs in subspecialty care is consistent with the fact that two of these counties (Cerro Gordo and Des Moines) are home to a rural referral hospital (Mason City and West Burlington, respectively). These counties had a high number of primary care APNs (Index = 143) and primary care physicians (Index = 118).

In summary, primary care PAs were present in proportional or higher than proportional numbers in three of the six rural categories of RUCC. For one additional non-metro category (mid-size, not adjacent to a metro area), the Index was 95. The only rural RUCC category where primary care PAs were present in small numbers compared to the population (Index of 61 in non-adjacent, metro area > 20K), there was a relatively large number of physicians and APNs practicing in primary care.

### Co-location patterns for primary care PAs

As mentioned above, policy analysts have suggested that multi-provider teams of primary care physicians, PAs and APNs could meet the increasing needs for primary care [3,4]. To provide a benchmark measure of such teams of primary care providers in Iowa, we examined the co-location patterns of primary care PAs, APNs and physicians in 2015.

We found that primary care PAs were working in 269 separate worksites. The average number of primary care PAs was 1.57 (Median = 1). A total of 47 primary care PAs (17%) were solo practitioners and an additional 10 worksites were populated only by primary care PAs. There were 96 sites consisting of primary care PAs and physicians only (36%) but only 18 sites staffed solely by primary care PAs and APNs (7%). This is disparity is likely due to the supervision requirements for PAs. Finally, a total of 98 sites were staffed by all three primary care providers (36%).

### HPSA location

**Fig 5:**
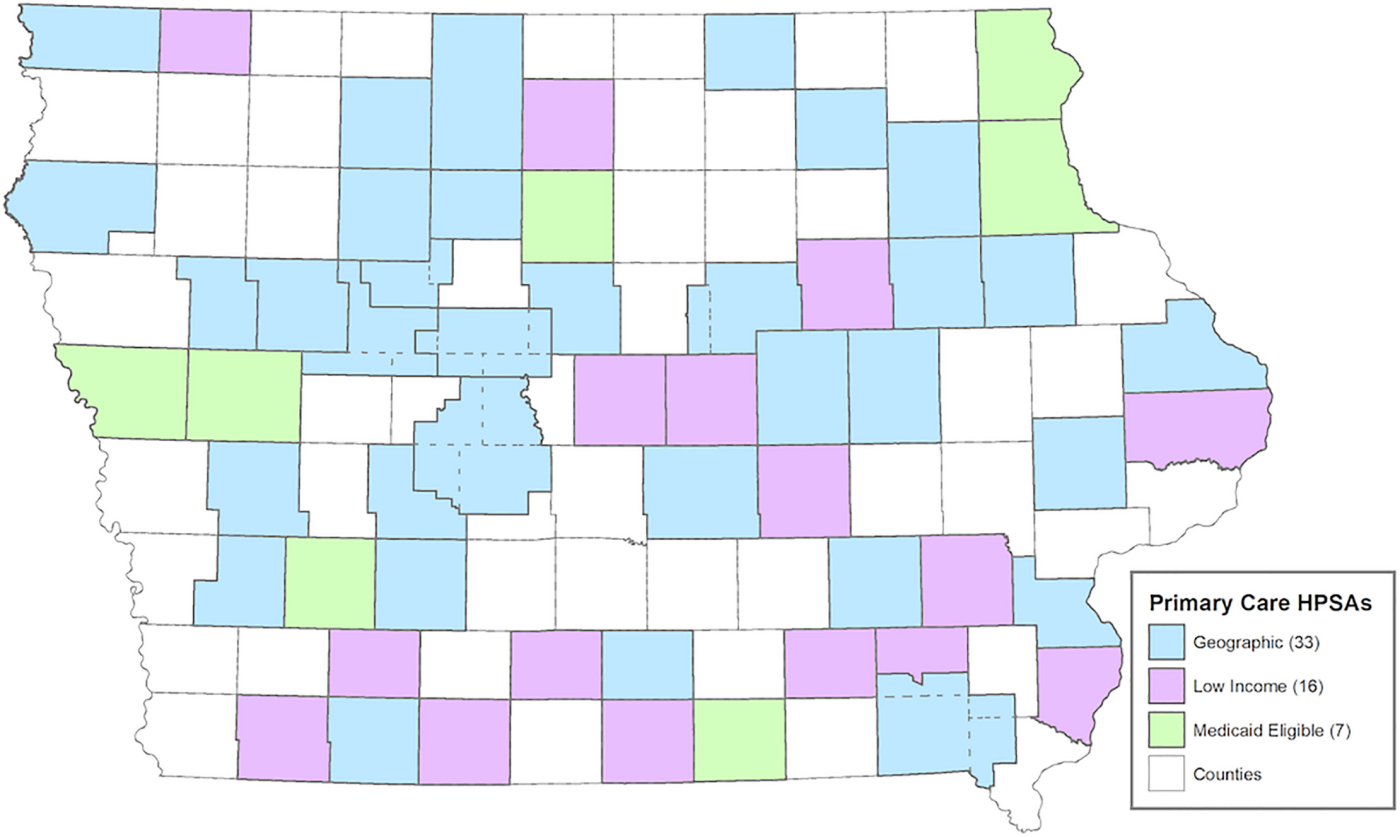
Health professional shortage areas for primary care in Iowa (2015).

As noted above, a high number (61) of Iowa’s 99 counties have been designated in whole (45) or in part (16) as a Health Professional Shortage Area (HPSA) for primary care (Fig 5). The locations of all PAs in primary care (family medicine, general internal medicine, general pediatrics, and obstetrics/gynecology) were coded with respect to a HPSA designation for the county (whole or in part) and/or worksite (e.g., rural health clinic).

There are 11 counties that do not have a practicing PA in 2015 (This figure has fallen from the 28 counties in 1995 that did not have a practicing PA). Of these 11 counties, five are designated at the county level as HPSAs and 2 are considered in part as being a HPSA for primary care. Four counties in Iowa without PAs are not considered HPSAs.

In the 40 counties (with a practicing PA) designated in whole as HPSAs for primary care, a total of 212 PAs were employed in 2015. Of these, 136 were in primary care. An additional 30 primary care PAs were identified as working in HPSAs in 8 additional counties. Using worksite data, an additional 22 primary care PAs were employed in HPSA designated sites such as rural health clinics, community health care centers and correctional facilities. The total number of primary care PAs working in HPSA geographic locations or designated sites was 188 in 2015. This represents 44% of the primary care PA workforce in Iowa and 22% of the entire PA workforce. By comparison, approximately 29% of primary care physicians practice within primary care HPSAs as do 40% of primary care APNs.

## Discussion

Like many states, Iowa has experienced a high level of growth in its PA workforce over the last 20 years. Given the national statistics on practice focus (primary v. subspecialty care), it is noteworthy that 56% of Iowa’s PA workforce is in primary practice. The wide geographic distribution of practice locations suggests that the state relies very heavily on PAs to deliver primary care to its citizens across the state.

In contrast to the national workforce, Iowa’s PA workforce is older. Given that the average retirement age in Iowa has been 64, Iowa faces a challenge to recruit and retain almost 30% of its entire PA workforce in the next 15 years.

The Iowa PA workforce has become more female over time (58% in 1995 compared to 71% in 2015). This trend follows the pattern of the increasing feminization of the national PA workforce [22]. However, it is important to note that—at the national level–the rural PA workforce is less female (66.5%) than the urban (71.2%) PA workforce [23]. In 2015, a high proportion of Iowa’s PAs were practicing in rural areas. However, if the location choices of new female PAs follow national trends, there may be fewer choosing rural areas, especially given the new opportunities in subspecialty fields. This represents another key challenge to the state as it attempts to provide access to primary care to it rural residents.

Recruitment of PAs new to Iowa may be a challenge. Currently, about half of the workforce trained in Iowa. Competition for graduates of programs in other states may increase as the demand for PAs in both primary and subspecialty care grows nationwide.

One possible strategy would be to improve the retention of Iowa-trained PAs. Currently, about 25% of PAs trained in Iowa end up in the local workforce. Despite the increased supply provided by new PA training programs, this historical retention rate may not be sufficient to meet the demands due to shifts in care models and retirements/relocations. A statewide strategy is needed to recruit PAs into primary care, especially in rural or underserved areas as well as to retain PAs already practicing in the state.

Reflecting the rural nature of Iowa, a higher proportion of Iowa’s PA workforce was employed in nonmetropolitan counties (~30%) compared to the national workforce (15%) in 2015. Furthermore, they have a large presence in the more isolated non-metro counties with the smallest urban populations (< 2,500). Primary care APNs are also well-represented in these counties.

Iowa has many counties (and worksites) designated as HPSAs. To meet the needs of these underserved populations, a large number of PAs provide primary care in those very locations (n = 188). This number represents 44% of the primary care PA workforce in Iowa. While there are no national figures for comparison, it seems apparent that Iowa depends heavily on its PA workforce to provide primary care to its most vulnerable citizens.

### Limitations

While the data on the PA workforce in Iowa is complete and continuously available since 1995, the findings reported here may be limited to Iowa due to its particular geography (multiple large rural cities), practice regulations and insurance environment among other factors. For example, in addition to physicians, Iowa State Code recognizes PAs, APNs and Chiropractors as primary care providers (§135.157), In addition, APNs are not required to be supervised by physicians as are PAs. This may alter the attractiveness of PAs as providers in some circumstances where physician supervision is more difficult.

Determining which APNs work in primary care is a major challenge [24]. Using the sole criteria of education, the number of APNs who could provide primary care is 1404. Once we controlled for practice setting/role (administration, hospitalist, urgent care) as well as specialty of the worksite (cardiology, etc.), the total primary care APN workforce was reduced to 857 (61%). Unlike states such as North Carolina, APNs in Iowa who are practicing primary care are not required to be under physician supervision. Thus, the methods used in other studies [24] to identify APNs in primary care is not feasible in Iowa.

A final limitation arises from the dynamic nature of any workforce. Like other professionals, PAs are highly mobile and some proportion are changing jobs over any given time period. Any cross-sectional analysis is an incomplete “snap shot” of the true state of the workforce. Due to a time lag in reporting/surveying, a PA listed with one specialty in our data extracted on 12/31/2015 may be actually employed at another facility and practicing under a different type of physician. Even with twice-yearly contact of every employer of PAs in the state of Iowa, there can be misclassifications of PAs. However, a detailed review of our data found very few such issues (12 out of 837 PAs or 1.4%).

## Conclusions

Iowa’s PA workforce has grown considerably over the past 20+ years and a high proportion is practicing in primary care. Furthermore, many of the PAs in primary care are serving in areas or institutions considered HPSAs for primary care. Future demand for PAs in subspecialty care will increase competition for PAs and may leave rural and other underserved areas of Iowa with reduced access to primary care. While the supply of PAs being trained in Iowa is expected to increase, the low level of retention in the state may be of concern. The practice and location choices of Iowa’s PA workforce should be monitored regularly to ensure that the primary care needs of Iowa’s most vulnerable populations are being met.

## Supporting information

S1 Table Yearly attrition of physician assistants in Iowa: 1995–2015. Iowa Health Professionals Inventory. Queried as of 12/31 of focal year.
(DOCX)

## References

[1] Bodenheimer T, Hoangmai HP. Primary care: Current problems and proposed solutions. Health Aff(Millwood) 2010;29(5):799–805 10.1377/hlthaff.2010.0026 20439864

[2] Iowa Department of Public Health. Iowa: Federal primary health care shortage designations. Created on 4/18/14. https://idph.iowa.gov/Portals/1/Files/RuralHealthPrimaryCare/ia_hpsa_primary_20140418.pdf (Retrieved 11/21/16).

[3] Green LV, Savin S, Lu Y. Primary care physician shortages could be eliminated through use of teams, nonphysicians, and electronic communication. Health Aff (Millwood). 2013; 32(1):11–19. 23297266 10.1377/hlthaff.2012.1086

[4] Auerbach DI, Chen PG, Friedberg MW, et al Nurse-managed health centers and patient-centered medical homes could mitigate expected primary care physician shortage. Health Aff (Millwood). 2013;32(11):1933–1941. 24191083 10.1377/hlthaff.2013.0596 PMC13477571

[5] Snyder J, Zorn J, Gjerde T, Burkhart J, Rosebrock L. The physician assistant workforce in Indiana: preparing to meet future health care needs. JAAPA. 2011 12;24(12):50, 53–7. 22315917 10.1097/01720610-201112000-00008

[6] Fraher EP, Morgan P, Johnson A. Specialty distribution of physician assistants and nurse practitioners in North Carolina. JAAPA. 2016 4;29(4):38–43. 10.1097/01.JAA.0000481402.98969.07 26953672

[7] Gadbois EA, Miller EA Tyler D, Intrator O. Trends in State Regulation of Nurse Practitioners and Physician Assistants, 2001 to 2010. Med Care Res Rev. 2015;72(2), 200–219. 10.1177/1077558714563763 25542195 PMC4730953

[8] Hass V. Physician assistants and nurse practitioners are not interchangeable. JAAPA. 2016;29(4):9–12. 10.1097/01.JAA.0000481408.81044.4e 26945275

[9] Rodgers GP, Conti JB, Feinstein JA, Griffin BP, Kennett JD, Shah S, Walsh MN, Williams ES, Williams JL. ACC 2009 survey results and recommendations: addressing the cardiology workforce crisis: a report of the ACC Board of Trustees Workforce Task Force. J Am Coll Cardiol. 2009;54:1195–208. 10.1016/j.jacc.2009.08.001 19761949

[10] Erikson C, Salsberg E, Forte G, Bruinooge S, Goldstein M. Future supply and demand for oncologists. J Oncol Pract. 2007;3(2): 62–65. 10.1200/JOP.0723601 PMC2793740 20859376

[11] Kim JSC, Cooper R, Kennedy DW, et al Otolaryngology–head and neck surgery physician work force issues: an analysis for future specialty planning. Otolaryngol Head Neck Surg. 2012;146:196–202. 10.1177/0194599811433977 24436481

[12] Mitchell KA, Spitz A. Use of advanced practice providers as part of the urologic healthcare team. Curr Urol Rep. 2015;16(9):62 10.1007/s11934-015-0535-5 26162306

[13] Dall TM, Gallo PD, Chakrabarti R, West T, Semilla AP, Storm MV. An aging population and growing disease burden will require a large and specialized health care workforce by 2025, Health Aff (Millwood). 2013;32(11):2013–2020. 24191094 10.1377/hlthaff.2013.0714

[14] Uhlman MA, Gruca TS, Erickson BA, MP32-03 Trends in the delivery of urologic procedural care by advanced practice providers, J Urol. 2016;193(4):e365.

[15] USDA Economic Research Service. 2013 Rural-Urban Continuum Codes. https://www.ers.usda.gov/data-products/rural-urban-continuum-codes.aspx (retrieved 11/15/2016).

[16] Dehn RW. The Distribution of Physicians, Advance Practice Nurses, and Physician Assistants In Iowa. The Journal of Physician Assistant Education. 2006 1 1;17(1):36–8.

[17] Health Resources and Services Administration Data Warehouse. https://datawarehouse.hrsa.gov/data/data.

[18] Kim HJ, Fay MP, Feuer EJ, et al: Permutation tests for joinpoint regression with applications to cancer rates. Stat Med. 2000(19):335–51. Erratum in: Stat Med. 2001 (20): 655. 10.1002/(sici)1097-0258(20000215)19:3<335::aid-sim336>3.0.co;2-z 10649300

[19] National Commission on Certification of Physician Assistants. (2015a). 2014 Statistical profile of certified physician assistants. https://www.nccpa.net/news/2015-statistical-profile-on-certified (retrieved 10/12/2017).

[20] American Academy of Physician Assistants. National physician assistant census report. research and technology services. AAPA Web site. http://www.aapa.org/uploadedFiles/content/Common/Files/National_Final_with_Graphics.pdf. Published 2009 (retrieved 11/29/2016).

[21] United States Census Bureau / American FactFinder. “B01003: Total Population” 2011–2015 American Community Survey 5-Year Estimates. U.S. Census Bureau’s American Community Survey Office, 2015. Web. 22 August 2018 <http://factfinder2.census.gov>.

[22] Lindsay S. The feminization of the physician assistant profession. Women & health. 2005 41(4), 37–61. 16260413 10.1300/J013v41n04_03

[23] Larson EH, Adrilla CHA, Morrison C, et al. Which physician assistant training programs produce rural PAs? A national study. Policy Brief #154. Seattle, WA: WWAMI Rural Health Research Center, University of Washington; http://depts.washington.edu/fammed/rhrc/wp-content/uploads/sites/4/2016/02/RHRC_PB154_Larson.pdf. Accessed August 27, 2018.

[24] Spetz J, Fraher E, Li Y, Bates T. How many nurse practitioners provide primary care? It depends on how you count them. Med Care Res Rev. 2015 6;72(3):359–75. 10.1177/1077558715579868 25854959

