# Supplementary material for: The workforce trends of physician assistants in Iowa (1995-2015)
Source: PLoS One. 2018 Oct 8;13(10):e0204813. doi: 10.1371/journal.pone.0204813 (PMC6175273; doi:10.1371/journal.pone.0204813)
Supplement: S1 Table — Iowa Health Professionals Inventory. Queried as of 12/31 of focal year. (DOCX) [file pone.0204813.s001.docx]

**Yearly Attrition of Physician Assistants in Iowa: 1995-2015^a^**

| Year | Retirement | Relocation | Death | Health | Training | Inactive | Misc. | Total |
| --- | --- | --- | --- | --- | --- | --- | --- | --- |
| 1995 | - | 1 | - | - | - | - | 2 | 3 |
| 1996 | - | 5 | - | - | - | - | 0 | 5 |
| 1997 | - | 5 | - | - | - | 1 | 4 | 10 |
| 1998 | - | 9 | - | 1 | 2 | 4 | 9 | 25 |
| 1999 | 1 | 6 | - | - | - | 3 | 10 | 20 |
| 2000 | 3 | 13 | - | - | 1 | 2 | 4 | 23 |
| 2001 | 1 | 27 | - | - | - | 6 | 7 | 41 |
| 2002 | 3 | 3 | - | 1 | 1 | 3 | 14 | 25 |
| 2003 | 3 | 8 | - | - | - | 5 | 15 | 31 |
| 2004 | 1 | 14 | 1 | 1 | 2 | 4 | 17 | 40 |
| 2005 | - | 10 | - | 1 | 1 | 2 | 14 | 28 |
| 2006 | 2 | 15 | - | 1 | 2 | 3 | 11 | 34 |
| 2007 | 2 | 20 | - | - | 1 | 5 | 11 | 39 |
| 2008 | 2 | 20 | 1 | 1 | - | 3 | 21 | 48 |
| 2009 | 3 | 13 | - | 1 | - | 3 | 11 | 31 |
| 2010 | 9 | 16 | - | 2 | - | 5 | 8 | 40 |
| 2011 | 7 | 29 | - | - | - | 4 | 10 | 50 |
| 2012 | 1 | 16 | 1 | - | - | 4 | 17 | 39 |
| 2013 | 8 | 13 | 2 | - | 2 | - | 14 | 39 |
| 2014 | 9 | 8 | 1 | - | - | 8 | 14 | 40 |
| 2015 | 8 | 22 | 0 | 2 | 0 | 3 | 38 | 73 |
| Total | 63 | 273 | 6 | 11 | 12 | 68 | 251 | 684 |
| **Annual Average** | 3.0 | 13.0 | 0.3 | 0.5 | 0.6 | 3.2 | 12.0 | 32.6 |
| **%age of Total** | 9% | 40% | 1% | 2% | 2% | 10% | 36% | 100% |
|  |  |  |  |  |  |  |  |  |
